# Maternal GALNT2 Variations Affect Blood Pressure, Atherogenic Index, and Fetal Growth, Depending on BMI in Gestational Diabetes Mellitus

**DOI:** 10.3389/fendo.2021.690229

**Published:** 2021-06-29

**Authors:** Linbo Guan, Ping Fan, Xinghui Liu, Mi Zhou, Yujie Wu, Rui Liu, Yu Liu, Huai Bai

**Affiliations:** ^1^ Laboratory of Genetic Disease and Perinatal Medicine and Key Laboratory of Birth Defects and Related Diseases of Women and Children of the Ministry of Education, West China Second University Hospital, Sichuan University, Chengdu, China; ^2^ Department of Obstetrics and Gynecology, West China Second University Hospital, Sichuan University, Chengdu, China; ^3^ Division of peptides related with human disease, West China Hospital, Sichuan University, Chengdu, China; ^4^ Department of Biochemistry and Molecular Biology, West China School of Preclinical and Forensic Medicine, Sichuan University, Chengdu, China

**Keywords:** GALNT2 gene, polymorphism (SNP), gestational diabetes mellitus, obesity, atherometabolic traits, fetal growth

## Abstract

**Background:**

GALNT2 is a GalNAc transferase that regulates serum lipid fractions, insulin signaling, and lipogenesis. Genetic variants are implicated in the pathogenesis of gestational diabetes mellitus (GDM). The objective of this study was to investigate the association of GALNT2 rs2144300 and rs4846914 single nucleotide polymorphisms (SNPs) with the risk of GDM and related traits.

**Methods:**

Two SNPs were genotyped, and clinical and metabolic parameters were determined in 461 GDM patients and 626 control subjects. Genetic associations with related traits were also analyzed.

**Results:**

The genotype distributions of the two SNPs in GDM patients were similar to those in normal controls. However, significant differences were noted across the three groups of genotypes with respect to the examined variables in subjects in a BMI-dependent manner. The rs4846914 and rs2144300 SNPs of GALNT2 were significantly associated with systolic blood pressure and/or diastolic blood pressure levels in nonobese GDM patients and atherogenic index (AI) in overweight/obese GDM patients. The rs4846914 SNP was also associated with fetal growth in overweight/obese GDM patients, and apo A1 and pregnancy weight gain in overweight/obese control women (all P<0.05).

**Conclusions:**

The two polymorphisms in the GALNT2 gene are associated with variations in blood pressure, atherogenic index, and fetal growth in GDM, depending on BMI, but not with GDM. Our findings highlight a link between related phenotypes in GDM mothers and their fetuses and the genetic components.

## Introduction

Gestational diabetes mellitus (GDM) is the most common metabolic disorder of pregnancy and is defined as any degree of glucose intolerance that begins or is first recognized during pregnancy (1). The prevalence of GDM varies worldwide and among different racial or ethnic groups (1, 2). The reported prevalence of GDM in pregnant Chinese women is 14.8% (2). The increased incidence of risk factors such as obesity, sedentary lifestyle, and older maternal age has led to an increased prevalence of GDM globally in recent years (1, 2). GDM can seriously harm both the mother and the fetus by increasing the risk of preeclampsia and macrosomia (1). GDM is also associated with an increased risk of metabolic-related disorders, such as type 2 diabetes mellitus, metabolic syndrome, and cardiovascular events in future life (1, 3). GDM might be related to genetic factors (4), dyslipidemia (5), oxidative stress (6), and inflammation (7).

It has been proposed that GDM has a complex, multifactorial etiology, in which a variety of predisposing genes interact with environmental factors to cause the disease (8). Although significant efforts have been made in recent years to identify the genes responsible for the risk of GDM, and many genes have been identified (9), the results are not complete.

The N-acetylgalactosaminyltransferase 2 (GALNT2) gene is located on the human chromosome region of 1q41-q42 and encodes a glycosyltransferase 2 protein, the widely expressed enzyme involved in regulating the initial step of mucin O-glycosylation. This enzyme has been found to affect serum HDL-C and TG levels in various studies in both humans and animal models (10). *GALNT2* is also reported to have a role in insulin sensitivity and glucose homeostasis in rodents (11) and to be a new potential modulator of cellular insulin action (12). It also plays a modulating role in adipogenesis and related cellular phenotypes (13), and influences the malignant behavior of several cancers (14).

Since *GALNT2* has demonstrated roles in various GDM-related traits such as lipid fractions, obesity-related phenotypes, and insulin sensitivity, it could be a strong candidate gene for predisposing effects of GDM. The rs2144300 and rs4846914 SNPs have been found to be associated with variations in blood lipid and cholesterol levels in genome-wide association studies (GWAS). These SNPs located within intron 1 of *GALNT2* have been demonstrated to be associated with HDL-C levels in various populations, as in European (15, 16), Mexican, and Japanese populations (17, 18). The two leading GWAS variants present perfect linkage disequilibrium (LD; r^2^ = 1). The rs4846914 SNP is also reported to be associated with triglyceride (TG) levels and lipoprotein size or fraction (19) as well as with a high risk of coronary heart disease (20). We speculated that there might be a relationship between *GALNT2* SNPs and the risk and/or related traits or phenotypes of GDM.

To date, there is little information concerning the possible relationship between GALNT2 polymorphisms and GDM. In this study, we evaluated the genotype distribution of *GALNT2* rs2144300 and rs4846914 SNPs in healthy pregnant women and women with GDM in a well-characterized Chinese population using relatively large sample sizes (461 cases and 626 controls). We also investigated whether the two SNPs were associated with the risk of GDM and related traits in our study subjects.

## Materials and Methods

### Study Subjects

This was a case-control study of 461 women with GDM and 626 women with uncomplicated pregnancies. The subjects were recruited from the Department of Obstetrics and Gynecology of West China Second University Hospital between 2013 and 2019. The study was carried out in accordance with the Declaration of Helsinki. Written informed consent was obtained from all participants. This study was approved by the Institutional Review Board of the West China Second University Hospital, Sichuan University (No. 2017–033).

All pregnant women at 24–28 gestational weeks underwent a routine 75g oral glucose tolerance test. The diagnosis of GDM was based on the guidelines of the International Association of Diabetes Pregnancy Study Groups (IADPSG), which specifies one or more of the following findings: fasting glucose ≥5.1 mmol/L, 1-h glucose ≥10.0 mmol/L, or 2-h glucose ≥8.5 mmol/L (21). Control women with uncomplicated pregnancies were recruited from the aforementioned department of the hospital during the same period. The inclusion criteria of the subjects were elective cesarean section and singleton pregnancy.

The exclusion criteria for the subjects were diabetes mellitus before pregnancy, chronic hypertension, any other pregnancy complications including preeclampsia and intrahepatic cholestasis of pregnancy; autoimmune, cardiac, renal, and hepatic diseases, and emergency cesarean delivery or multiple pregnancies.

Clinical and anthropometric variables, including systolic blood pressure (SBP), diastolic blood pressure (DBP), and body mass index (BMI, kg/m^2^) were measured or assessed. Normal weight and overweight/obesity was defined as BMI <25 and ≥25kg/m^2^, respectively.

Blood samples of the study subjects were obtained on the morning of the elective cesarean section after an overnight fast. The blood samples were kept on ice and centrifuged at 1500×g for 15 min at 4°C. The plasma and serum aliquots were stored at −80°C for later analysis. The collection and separation of samples were completed within 4 h.

### DNA Extraction and Genotyping

Genomic DNA was isolated from 500 μL of peripheral blood according to the method of Erlich (22). Genotypes for rs2144300 and rs4846914 polymorphisms were determined by PCR-RFLP and the Taqman real-time PCR allelic discrimination assay, respectively, as described previously (23).

The above genotyping results (greater than 30% of DNA samples) were confirmed by a direct DNA sequencing approach.

### Analysis of Metabolic Parameters

Plasma insulin levels were measured by chemiluminescence assays (IMMULITE 2000; Diagnostic Products Corporation, Los Angeles, CA, USA). Plasma glucose was measured using the glucose oxidase technique (Roche Diagnostics, Mannheim, Germany). The concentrations of plasma total cholesterol (TC), high-density lipoprotein cholesterol (HDL-C), low-density lipoprotein cholesterol (LDL-C), and triglycerides (TGs) were measured by an enzyme assay (Boehringer, Mannheim, Germany), and serum apoA1 and B levels were determined by polyethylene glycol-enhanced immunoturbidimetric assay (Siemens Healthcare Diagnostics, Inc.) using a Hitachi 7600–010 automatic analyzer. The intra- and interassay coefficients of variation for all measurements were less than 5% and 10%, respectively.

Homeostatic model assessment of insulin resistance (HOMA-IR) was determined as fasting glucose (mmol/L) × fasting insulin (μU/mL)/22.5 (24). The atherogenic index (AI) was calculated using the following equation: AI = [TC−(HDL-C)/(HDL-C)] (25). Non-HDL-C was calculated as TC minus HDL-C.

### Statistical Analysis

Data are presented as mean ± SD. Allele frequencies of GALNT2 polymorphisms were estimated by gene counting. Hardy–Weinberg equilibrium was tested in cases and controls using the chi-square test. Allele and genotype frequencies were compared between cases and controls using chi-square analysis. Differences in variables were evaluated by independent-sample t-tests between the GDM and control subjects. To evaluate the effect of GALNT2 polymorphisms on the variation in quantitative variables, ANOVA was carried out. Adjustment for age and BMI was performed by analysis of covariance. All statistical analyses were performed using the Statistical Package for Social Sciences version 13.0 for Windows (SPSS, Chicago, IL, USA).

## Results

### Clinical and Biochemical Characteristics

As shown in **Table 1**, prepregnancy BMI and delivery BMI were significantly higher, and pregnancy week at delivery and parity were significantly lower in the GDM group than in the control group (**Table 1**). Women with GDM also had significantly higher fasting insulin and glucose concentrations, HOMA-IR, TG, TG/HDL-C ratios, and lower LDL-C and apo A1 levels compared with the control women after adjusting for the difference in age and BMI at delivery (**Table 1**). These results are generally consistent with the clinical and metabolic profiles frequently presented in GDM subjects, as observed in our recent study (26).

**Table 1:** Clinical and biochemical characteristics in patients with GDM and control women.

	GDM (n = 461)	Control (n=626)	P values
**Clinical characteristics**			
Age (years)	34.64 ± 4.28	34.81 ± 4.3	0.529
Pregnancy week at delivery	29.04 ± 0.91	29.24 ± 0.97	0.001
Parity	1.60 ± 0.52	1.67 ± 0.53	0.027
Prepregnancy BMI (kg/m^2)^	22.22 ± 3.23	20.95 ± 2.78	0.000
Weight gain during pregnancy(kg)	11.84 ± 4.29	14.11 ± 4.65	0.000
Delivery BMI (kg/m^2)^	26.91 ± 3.57	26.49 ± 2.93	0.035
SBP (mmHg)	115.28 ± 11.42	114.3 ± 10.08	0.133
DBP (mmHg)	72.75 ± 9.19	71.93 ± 8.48	0.128
Neonatal birth height (cm)	49.56 ± 2.91	49.84 ± 1.88	0.053
Neonatal birth weight (g)	3361.65 ± 467.04	3424.8 ± 1241.17	0.297
**Metabolic profile**			
Fasting Ins (pmol/L)	85.14 ± 57.25	71.31 ± 39.64	0.000
Fasting Glu (mmol/L)	4.42 ± 0.92	4.16 ± 1.00	0.000
HOMA-IR	3.68 ± 10.29	2.36 ± 3.38	0.008
Triglycerides (mmol/L)	3.88 ± 1.67	3.62 ± 1.46	0.006
TC (mmol/L)	5.98 ± 1.31	6.02 ± 1.18	0.559
HDL-C (mmol/L)	1.98 ± 0.47	2.00 ± 0.45	0.566
LDL-C (mmol/L)	2.98 ± 0.99	3.14 ± 1.05	0.011
non-HDLC (mmol/L)	4.09 ± 1.20	4.08 ± 1.11	0.910
Atherogenic index (AI)	2.11 ± 0.67	2.05 ± 0.68	0.309
TG/HDL-C	2.15 ± 1.15	1.88 ± 0.91	0.001
Apo A1 (g/L)	2.29 ± 0.37	2.42 ± 0.46	0.000
Apo B (g/L)	1.18 ± 0.26	1.16 ± 0.28	0.429
Apo B/apo A1 ratio	0.57 ± 0.14	0.55 ± 0.16	0.110

Values are present as mean ± SD.

### GALNT2 Genotype and Allele Frequencies

Genotypes of the rs2144300 and rs4846914 polymorphisms were in Hardy–Weinberg equilibrium in both GDM patients and control groups. The frequency data are presented in **Table 2**. The C and T allele frequencies of *GALNT2* at the rs2144300 site were 82.2% and 17.8%, respectively, in the GDM group, and 80.9% and 19.1%, respectively, in the normal control group. The G and A allele frequencies of *GALNT2* at the rs4846914 site were 75.7% and 24.3%, respectively, in the GDM group, and 75.8% and 24.2%, respectively, in the control group. The genotype and allele frequencies of the two polymorphisms in the GDM subjects were not different from those in the normal controls (P>0.05).

**Table 2:** Genotype and allele frequency for the rs2144300 and rs4846914 SNPs of the GALNT2 gene in women with GDM and control subjects.

	GDM (n = 461)	Control (n = 626)	*p*
**rs2144300**			
Genotype			0.178
CC	66.8% (308)	66.3% (415)	
CT	30.8% (142)	29.2% (183)	
TT	2.4% (11)	4.5% (28)	
Allele			0.440
C	82.2% (758)	80.9% (1013)	
T	17.8% (164)	19.1% (239)	
**rs4846914**			
Genotype			0.926
GG	58.8% (271)	58.5% (366)	
GA	33.8% (156)	34.6% (217)	
AA	7.4% (34)	6.9% (43)	
Allele			0.960
G	75.7% (698)	75.8% (949)	
A	24.3% (224)	24.2% (303)	

Numbers in parentheses indicate number of subjects with each genotype or number of alleles of each type.

In addition, when GDM patients and control subjects were further divided into overweight/obese and nonobese subgroups, no significant differences in genotype or allele frequencies between overweight/obese and nonobese patients or between overweight/obese and nonobese control subjects were observed at these sites (**Table 3**).

**Table 3:** Genotype and allele frequency for the rs2144300 and rs4846914 SNPs of the GALNT2 gene in women with GDM and control subjects divided into overweight/obese and nonobese subgroups.

	GDM			Control		
	Overweight/Obese (n = 330)	Non-obese (n = 131)	*p*	Overweight/Obese (n = 452)	Non-obese (n = 174)	*p*
**rs2144300**						
Genotype			0.455			0.934
CC	68.5% (226)	61.6% (82)		65.9% (298)	67.2% (117)	
CT	29.4% (97)	34.3% (45)		29.7% (134)	28.2% (49)	
TT	2.1% (7)	3.1% (4)		4.4% (20)	4.6% (8)	
Allele			0.222			0.818
C	83.2% (549)	79.8% (209)		80.8% (731)	81.3% (283)	
T	16.8% (111)	20.2% (53)		19.2% (174)	18.7% (65)	
**rs4846914**						
Genotype			0.774			0.611
GG	57.9% (197)	56.5% (74)		58.0% (262)	59.8% (104)	
GA	33.3% (110)	35.1% (46)		35.6% (161)	32.2% (56)	
AA	7.0% (23)	8.4% (11)		6.4% (29)	8.0% (14)	
Allele			0.459			0.974
G	76.4% (504)	74.0% (194)		75.8% (685)	75.9% (264)	
A	23.6% (156)	26.0% (68)		24.2% (219)	24.1% (84)	

Numbers in parentheses indicate number of subjects with each genotype or number of alleles of each type.

### Effect of GALNT2 SNPs on Clinical and Metabolic Parameters in Overweight/Obese and Nonobese Subgroups

There were no significant differences in clinical and metabolic parameters across the three genotypes at rs4846914 and rs2144300 SNPs in both GDM patients and the normal control groups (**Supplementary Tables 1** and **2**).

Since the GALNT2 gene plays a role in lipogenesis in fat tissue (obese phenotype), we further analyzed the effects of GALNT2 gene polymorphisms on clinical and metabolic parameters in GDM patients and control subjects stratified into overweight/obese (BMI≥25 kg/m^2^) and nonobese (BMI<25 kg/m^2^) subgroups. We found that the AA homozygotes at the rs4846914 site in the nonobese GDM group had higher SBP and DBP levels than the GG homozygotes (P=0.035 and P=0.005, respectively) (**Table 4**). In addition, in the overweight/obese GDM group, the GA heterozygotes had a higher atherogenic index (P=0.011), and the AA homozygotes had lower neonatal birth weight and height (P=0.038 and P=0.013, respectively) than GG homozygotes (**Table 4**). The logistic regression analysis with a binary outcome of macrosomia (with and without macrosomia) showed that the birth weight (macrosomia) correlated positively with prepregnancy BMI, GWG, maternal BMI, and triglycerides (TG) levels (OR=4.337, 95% CI=1.230~15.297; OR=1.974, 95% CI=1.234~3.158; OR=0.297, 95% CI=0.089~0.972, and OR=1.431, 95% CI=1.093~1.874. P<0.01 ~ P<0.05), but not correlated with the genetic variants of the rs2144300 and rs 4846914 SNPs (OR=1.719, 95% CI=0.427~6.915, P=0.446; OR=0.440, 95% CI=0.111~1.739, P=0.242).

**Table 4:** Clinical characteristics and metabolic profile of GALNT2 rs4846914 genotypes in overweight/obese and non-obese GDM patients.

	Overweight/Obese GDM			Non-obese GDM		
	GG (197)	GA (110)	AA (23)	GG (74)	GA (46)	AA (11)
**Clinical characteristics**						
Age (years)	35.85 ± 3.11	34.75 ± 4.11	35.29 ± 4.15	36.08 ± 4.08	32.34 ± 4.54	33.44 ± 4.42
Gestation age (weeks)	39.08 ± 0.82	38.97 ± 0.90	39.17 ± 0.65	38.91 ± 1.25	39.18 ± 0.65	39.10 ± 0.75
Prepregnancy BMI (kg/m^2^)	23.58 ± 3.01	23.5 ± 3.65	23.3 ± 2.52	19.72 ± 1.07	18.81 ± 1.52	19.29 ± 1.39
Weight gain during pregnancy (kg)	11.29 ± 4.12	12.38 ± 4.50	12.25 ± 4.52	9.13 ± 4.22	11.54 ± 2.67	10.76 ± 3.85
Delivery BMI (kg/m^2^)	29.19 ± 6.43	28.43 ± 3.72	28.09 ± 2.29	23.39 ± 1.10	23.33 ± 1.34	23.55 ± 1.04
Neonatal birth height (cm)	49.83 ± 1.81	49.95 ± 1.62	49.00 ± 1.62^a,*^	49.28 ± 1.55	49.44 ± 1.97	49.25 ± 2.42
Neonatal birth Weight (g)	3358.02 ± 385.86	3366.44 ± 368.25	3194.38 ± 406.11^a,*^	3172.21 ± 352.45	3171.09 ± 349.97	3330.91 ± 411.96
SBP (mmHg)	114.69 ± 8.80	116.96 ± 11.33	116.19 ± 12.79	112.24 ± 9.41	113.79 ± 9.45	119.58 ± 10.87^a,*^
DBP (mmHg)	73.84 ± 9.13	73.05 ± 9.93	73.22 ± 9.21	70.69 ± 7.81	74.43 ± 9.41	79.17 ± 9.51^a,**^
**Metabolic profile**						
Fasting Ins (pmol/L)	89.24 ± 58.83	88.95 ± 56.12	101.61 ± 68.35	66.20 ± 45.06	81.14 ± 60.31	80.95 ± 55.38
Fasting Glu (mmol/L)	4.32 ± 0.52	4.47 ± 0.68	4.41 ± 0.79	4.67 ± 0.84	4.36 ± 0.96	4.37 ± 0.73
HOMA-IR	2.93 ± 2.16	3.18 ± 3.09	3.08 ± 3.74	3.55 ± 3.53	3.56 ± 4.50	2.53 ± 4.49
Triglycerides (mmol/L)	3.55 ± 1.16	4.12 ± 1.82	3.95 ± 1.72	3.30 ± 0.80	3.55 ± 1.25	3.92 ± 2.11
TC (mmol/L)	5.56 ± 0.76	5.94 ± 1.07	5.90 ± 1.21	6.19 ± 1.11	5.98 ± 0.92	6.15 ± 1.09
HDL-C (mmol/L)	1.90 ± 0.39	1.87 ± 0.44	1.97 ± 0.46	2.24 ± 0.71	2.06 ± 0.40	2.08 ± 0.54
LDL-C (mmol/L)	2.75 ± 0.68	2.94 ± 0.88	2.94 ± 0.93	3.19 ± 0.80	3.22 ± 1.68	3.07 ± 0.91
non-HDLC (mmol/L)	3.79 ± 0.68	4.14 ± 0.93	4.02 ± 1.06	4.14 ± 0.76	3.97 ± 0.93	4.40 ± 1.96
Atherogenic index	2.03 ± 0.64	2.34 ± 0.89^a,*^	2.07 ± 0.63	1.87 ± 0.64	2.02 ± 0.73	2.16 ± 0.72
TG/HDL-C	2.12 ± 0.91	2.72 ± 2.46	2.16 ± 1.05	1.58 ± 0.58	1.87 ± 0.78	2.11 ± 1.23
apoA1(g/L)	2.24 ± 0.27	2.30 ± 0.38	2.29 ± 0.37	2.31 ± 0.36	2.31 ± 0.37	2.32 ± 0.40
apoB (g/L)	1.09 ± 0.22	1.19 ± 0.26	1.17 ± 0.28	1.20 ± 0.23	1.16 ± 0.22	1.21 ± 0.26
apoB/apoA1 ratio	0.54 ± 0.13	0.57 ± 0.13	0.57 ± 0.15	0.54 ± 0.11	0.55 ± 0.13	0.59 ± 0.14

a compared with GG genotype carriers in the same group, *p < 0.05, **p < 0.01.

In the overweight/obese control group, GA heterozygotes at rs4846914 site had lower apo A1 levels and pregnancy weight gain than GG homozygotes (P=0.04 and P=0.039, respectively). Genotype-related effects were not observed in nonobese control subjects at the rs4846914 site (**Table 5**).

**Table 5:** Clinical characteristics and metabolic profile of GALNT2 rs4846914 genotypes in overweight/obese and non-obese control subjects.

	Overweight/Obese control			Non-obese control		
	GG (262)	GA (161)	AA (29)	GG (104)	GA (56)	AA (14)
**Clinical characteristics**						
Age (years)	35.25 ± 3.81	36.19 ± 7.1	35.69 ± 4.19	34.56 ± 4.37	34.23 ± 4.22	35.19 ± 2.25
Gestation age (weeks)	39.30 ± 0.90	39.19 ± 1.16	39.20 ± 0.90	39.31 ± 0.86	38.97 ± 0.95	39.24 ± 0.88
Prepregnancy BMI (kg/m^2^)	21.75 ± 2.77	22.1 ± 2.21	22.30 ± 3.15	18.66 ± 2.15	18.84 ± 1.37	18.71 ± 1.25
Weight gain during pregnancy (kg)	15.07 ± 5.03	14.01 ± 3.92^a,*^	14.29 ± 4.39	12.11 ± 3.12	12.36 ± 3.48	11.4 ± 2.95
Delivery BMI (kg/m^2^)	27.77 ± 2.02	27.64 ± 2.10	28.00 ± 3.34	23.36 ± 2.36	23.56 ± 1.06	23.11 ± 0.92
Neonatal birth height (cm)	49.68 ± 2.29	49.87 ± 1.67	50.04 ± 1.60	49.53 ± 1.86	49.31 ± 1.80	49.90 ± 1.67
Neonatal birth Weight (g)	3450.76 ± 1654.66	3360.42 ± 355.90	3415.66 ± 382.76	3277.62 ± 358.52	3211.81 ± 337.84	3239.00 ± 363.08
SBP (mmHg)	115.94 ± 10.41	114.37 ± 10.27	114.04 ± 9.63	112.11 ± 9.38	111.79 ± 9.88	112.48 ± 10.08
DBP (mmHg)	72.77 ± 7.86	72.21 ± 7.99	71.20 ± 7.36	71.92 ± 8.02	70.76 ± 11.66	71.43 ± 8.36
**Metabolic profile**						
Fasting Ins (pmol/L)	76.06 ± 40.79	69.31 ± 35.46	78.72 ± 41.08	59.47 ± 40.02	67.82 ± 38.99	77.02 ± 39.01
Fasting Glu (mmol/L)	4.21 ± 0.56	4.15 ± 0.59	4.18 ± 0.72	4.09 ± 0.71	4.15 ± 0.62	4.38 ± 0.49
HOMA-IR	2.22 ± 1.60	1.97 ± 1.22	2.16 ± 1.27	1.85 ± 2.48	1.79 ± 1.24	2.30 ± 1.48
Triglycerides (mmol/L)	3.79 ± 1.60	3.58 ± 1.49	3.64 ± 1.40	3.32 ± 1.14	3.56 ± 1.24	3.27 ± 1.00
TC (mmol/L)	6.03 ± 1.13	5.90 ± 1.16	5.70 ± 1.25	6.08 ± 1.21	6.42 ± 1.22	6.37 ± 1.27
HDL-C (mmol/L)	1.99 ± 0.41	1.91 ± 0.42	1.94 ± 0.53	2.05 ± 0.51	2.16 ± 0.46	2.22 ± 0.49
LDL-C (mmol/L)	3.12 ± 1.11	3.09 ± 0.95	2.85 ± 1.05	3.24 ± 0.93	3.46 ± 1.07	3.32 ± 1.17
non-HDLC (mmol/L)	4.10 ± 1.20	3.96 ± 0.95	3.75 ± 0.92	4.10 ± 0.97	4.30 ± 1.00	4.55 ± 1.26
Atherogenic index	2.10 ± 0.67	2.12 ± 0.66	1.85 ± 0.63	1.97 ± 0.75	2.02 ± 0.67	2.13 ± 0.54
TG/HDL-C	2.08 ± 0.99	1.93 ± 0.79	1.83 ± 0.94	1.66 ± 0.74	1.72 ± 0.81	1.67 ± 0.55
apoA1 (g/L)	2.47 ± 0.48	2.35 ± 0.42^a,**^	2.39 ± 0.38	2.39 ± 0.47	2.47 ± 0.42	2.53 ± 0.43
apoB (g/L)	1.16 ± 0.28	1.15 ± 0.26	1.11 ± 0.31	1.17 ± 0.28	1.25 ± 0.30	1.19 ± 0.33
apoB/apoA1 ratio	0.54 ± 0.16	0.55 ± 0.15	0.50 ± 0.18	0.55 ± 0.18	0.58 ± 0.15	0.63 ± 0.17

a compared with GG genotype carriers in the same group, *p < 0.05, **p < 0.01.

In addition, in the nonobese GDM group, CC, CT, and TT genotype carriers at the rs2144300 site showed a tendency of a genetic dose-dependent increase in SBP and DBP levels. However, only TT and CT carriers showed statistically significant differences in DBP levels (P=0.033 and P=0.007, respectively). In the overweight/obese GDM group, CT heterozygotes at the same site had higher AI than CC homozygotes (P=0.021) (**Table 6**). Genotype related effects on these parameters at the rs2144300 site were not observed in overweight/obese and nonobese control groups (data not shown).

**Table 6:** Clinical characteristics and metabolic profile of GALNT2 rs2144300 genotypes in overweight/obese and non-obese GDM patients.

	Overweight/Obese GDM			Non-obese GDM		
	CC (226)	CT (97)	TT (7)	CC (82)	CT (45)	TT (4)
**Clinical characteristics**						
Age (years)	35.23 ± 4.14	34.82 ± 4.07	36.67 ± 2.65	33.56 ± 4.36	32.55 ± 4.56	37.8 ± 4.27
Gestation age (weeks)	39.07 ± 0.81	38.99 ± 0.91	39.42 ± 0.58	38.93 ± 1.22	39.15 ± 0.71	39.10 ± 0.48
Prepregnancy BMI (kg/m^2^)	23.36 ± 2.57	23.45 ± 3.78	22.8 ± 2.08	19.28 ± 1.4	18.99 ± 1.52	19.67 ± 0.95
Weight gain during pregnancy (kg)	12.17 ± 4.63	12.23 ± 4.34	12.61 ± 2.23	10.64 ± 3.75	11.11 ± 3.18	10.30 ± 4.44
Delivery BMI (kg/m^2^)	28.13 ± 2.33	28.61 ± 4.85	27.78 ± 2.20	23.51 ± 1.09	23.34 ± 1.22	23.69 ± 1.41
Neonatal birth height (cm)	49.87 ± 1.79	49.68 ± 1.71	49.22 ± 1.20	48.78 ± 5.7	49.22 ± 2.02	50.00 ± 3.16
Neonatal birth Weight (g)	3431.59 ± 448.09	3382.73 ± 443.23	3301.11 ± 218.48	3212.56 ± 547.36	3208.44 ± 399.01	3662.00 ± 471.18
SBP (mmHg)	115.98 ± 12.63	115.96 ± 10.9	114.11 ± 10.65	112.58 ± 9.47	114.7 ± 9.67	119.6 ± 12.36
DBP (mmHg)	72.57 ± 9.36	72.68 ± 9.22	73.56 ± 9.32	70.98 ± 8.08	75.43 ± 9.79^a,**^	79.6 ± 7.67^b,*^
**Metabolic profile**						
Fasting Ins (pmol/L)	90.69 ± 60.64	87.03 ± 54.06	108.65 ± 60.49	66.78 ± 45.05	81.46 ± 60.46	75.21 ± 54.79
Fasting Glu (mmol/L)	4.44 ± 1.04	4.41 ± 0.77	4.46 ± 0.77	4.42 ± 0.81	4.32 ± 0.87	4.42 ± 0.86
HOMA-IR	4.13 ± 13.51	3.69 ± 7.92	3.44 ± 2.07	2.96 ± 5.09	3.01 ± 3.29	2.70 ± 2.23
Triglycerides (mmol/L)	3.94 ± 1.66	4.04 ± 1.54	3.19 ± 1.22	3.9 ± 2.08	3.49 ± 1.11	3.01 ± 0.78
TC (mmol/L)	5.88 ± 1.20	5.94 ± 1.01	5.55 ± 0.97	6.38 ± 1.96	5.96 ± 0.85	5.57 ± 1.32
HDL-C (mmol/L)	1.97 ± 0.46	1.91 ± 0.42	1.71 ± 0.33	2.08 ± 0.54	2.08 ± 0.45	1.95 ± 0.68
LDL-C (mmol/L)	2.93 ± 0.93	2.92 ± 0.83	2.93 ± 0.81	3.09 ± 0.90	3.25 ± 1.66	2.71 ± 0.65
non-HDLC (mmol/L)	3.99 ± 1.03	4.09 ± 0.91	4.29 ± 0.68	4.43 ± 1.92	3.93 ± 0.88	4.05 ± 0.61
Atherogenic index	2.05 ± 0.60	2.23 ± 0.71^a,*^	2.60 ± 0.81	2.18 ± 0.72	1.98 ± 0.70	1.88 ± 0.52
TG/HDL-C	2.05 ± 0.60	2.41 ± 1.43	2.30 ± 1.18	2.18 ± 0.72	1.74 ± 0.80	1.63 ± 0.50
apoA1(g/L)	2.28 ± 0.37	2.32 ± 0.36	2.15 ± 0.34	2.32 ± 0.41	2.29 ± 0.35	2.27 ± 0.42
apoB (g/L)	1.16 ± 0.28	1.18 ± 0.24	1.15 ± 0.32	1.22 ± 0.26	1.16 ± 0.21	1.08 ± 0.24
apoB/apoA1 ratio	0.56 ± 0.14	0.56 ± 0.12	0.65 ± 0.19	0.59 ± 0.14	0.54 ± 0.13	0.51 ± 0.09

^a,b^compared with CC genotype carriers in the same group, respectively, *p < 0.05, **p < 0.01.

## Discussion

Our results in a Chinese cohort living in Southwest China showed for the first time that both the rs4846914 and rs2144300 SNPs of *GALNT2* were associated with SBP and/or DBP levels in nonobese GDM patients, and AI in overweight/obese GDM patients, while the rs4846914 SNP was also associated with fetal growth in overweight/obese GDM patients, and apo A1 and pregnant weight gain in overweight/obese control women. These results provide evidence that the polymorphisms are related to blood pressure levels, atherogenic traits, and fetal growth in GDM patients, in addition to their relationship with metabolic and anthropometric parameters. These results establish a link between related phenotypes in GDM mothers and their neonates and genetic components.

An initial study on the *GALNT2* rs4846914 polymorphism was carried out using plasma HDL cholesterol and Mexican dyslipidemic study samples of a Caucasian population for triglyceride levels (17). The frequency of this polymorphic site was reported to be high in Mexican, Mexican-American, and European populations with G allele frequencies of 37%, 51%, and 43%, respectively. The G allele frequency of this polymorphism in Roma and Hungarian populations was also high, with G allele frequencies of 46.6% and 54.5%, respectively (27). In this study, we found that the G allele frequency in the Southwest Han Chinese population was 75.8%, which is higher than that reported in other populations or ethnic groups (18, 27). In addition, the distribution of T allele frequency in the rs2144300 SNP was 19.1% in the Han Chinese, which is in contrast with the higher frequency of 42.2% in American Caucasians (28).

The incidence of the allele frequencies of the rs4846914 and rs2144300 polymorphisms in the patients with GDM were not significantly different from those in the controls (P>0.05).

In the current study, nonobese GDM patients carrying the respective variant of the two SNPs showed significantly higher SBP and/or DBP levels compared with those carrying the respective wild-type variants; however, these differences were not observed in overweight/obese GDM patients. Clinical diabetes, including GDM, is considered an independent risk factor for cardiovascular complications. Women with GDM are at a higher risk of hypertensive disorder or preeclampsia. One study by transcriptomics showed in the placenta, a highly expressed pro-inflammatory pattern mainly of endothelial factors, reflecting chronic inflammation with signs of major vascular dysfunction (29). Another study found increased levels of an array of pro-inflammatory molecules (30, 31), as well as a pro-inflammatory pattern of upregulated adipokines and diminished anti-inflammatory adiponectin in adipose tissue from women with GDM (32). This pro-inflammatory milieu, together with dysregulated secretion of placental factors and/or β-cell injury, could trigger not only metabolic but also cardiovascular abnormalities including hypertension in women with GDM and their offspring (32).

It is unclear why overweight/obese women with GDM did not show genotype-related variations in blood pressure. It has been proposed that obesity can trigger more complex pathways related to the cardiovascular system in women with GDM. These factors may affect the genotype-mediated role in blood pressure and, therefore, possibly mask the genotype-related effect on blood pressure in overweight/obese patients.

In the present study, we also found an association of rs4846914 and rs2144300 sites with AI in overweight/obese GDM patients. GA and CT heterozygotes in the overweight/obese GDM group showed higher AI values than GG and CC homozygotes, respectively (GA vs. GG: 2.34 ± 0.89 vs. 2.07 ± 0.63, P=0.011; CT vs. TT: 2.23 ± 0.71 vs. 2.05 ± 0.60, P=0.021). Demirel et al. found that AI was correlated with BMI and leptin levels in PCOS subjects. Leptin was the most important factor affecting AI in multiple regression analysis (25). These authors proposed that leptin, as well as other adipocytokines, potentially affect atherogenesis and might be linked to higher AI values. We speculate that higher AI values might also be related to some adipocytokines in overweight/obese GDM women and modified by the genetic components. In addition, the genotype-related elevation of AI in this study may also reflect the lipid variations in overweight/obese GDM patients related to genetic factors. These data support the notion of obese GDM at higher CAD risk and suggest that the genetic elements are associated with the atherogenic risk marker as AI measure in such patients, being potentially linked to the elevated risk of cardiovascular disease (CVD) in these women.

Interestingly we observed a genotype association with fetal growth, neonatal birth weight, and height measurements in overweight/obese women with GDM. Previous genome-wide association studies of birth weight identified a variant in the ADCY5 (rs9883204) associated with birth weight and type 2 diabetes, and another variant, near CCNL1 (rs900400), associated with no obvious link to adult traits (33). In an expanded genome-wide association meta-analysis and follow-up study of birth weight, the authors found a new association with birth weight at 4 loci (index SNPs: rs1042725 in HMGA2, rs724577 in LCORL, rs 1801253 in ADRB1, and rs4432842 on chromosome 5q 11.2) (34). The present results may link the polymorphism as one of the fetal birth weight related genes in the human population. Alternatively, this relationship might be driven by specific maternal metabolism. Our additional logistic regression analysis with a binary outcome of macrosomia demonstrated that the birth weight (macrosomia) correlated positively with pre-pregnancy BMI, GWG, maternal BMI, and TG levels (P<0.05 ~ P<0.01), but not correlated with the gene polymorphisms. This issue needs to be addressed with an expanded sample size further.

It has been proposed that size at birth is determined by both genetic factors and the intrauterine environment, as determined by maternal plasma glucose, obesity, and other factors (35, 36). One study involving up to 11307 mother-child pairs showed that the associations were driven by the fetal genotype (34). Of note, throughout the cellular processes of gametogenesis and fertilization, the fetal genotype is correlated with the maternal genotype (r≈0.5). We speculate that the present association of the rs4846914 variant with fetal growth may be associated with the fetal genotype as well because the maternal genotype may be correlated with the fetal genotype.

Gestational weight gain (GWG) is a modifiable risk factor for obesity in women, which contributes to the obesity epidemic and disease risk. Excessive GWG and prepregnancy body mass index (BMI) are known contributors to postpartum weight retention. For example, over 50% of pregnant women in the United States gain more weight than the IOM recommends (37) and therefore at an increased risk of long-term obesity, which is associated with adverse health consequences such as hypertension, cardiovascular disease, diabetes mellitus, and osteoarthritis (38). GWAS and candidate gene approaches have shown some gene variants linked to GWG. The present results showing an association between the rs4846914 SNP and pregnancy weight gain in overweight/obese control women may provide additional support for the scope of GWG-related genes in the human population. A lack of association between the studied genetic variants of the GALNT2 gene and GWG in the GDM patients could be explained by a predominant impact of a dietary treatment on the GWG in this group.

The GALNT2 rs4846914 SNP was first reported to be associated with variations in serum HDL-C and TG levels in Mexican dyslipidemic study samples, including Mexican, Mexican American, and European populations, using a GWAS approach (17). In this study, overweight/obese control subjects carrying the GA genotype showed lower apoA1 levels compared with those carrying the AA genotype. This result, in general, is in line with the above report, as apoA1 is the major apolipoprotein component of HDL-C particles and plays an important role in HDL-C function. However, we were unable to find similar genotype-related changes in apoA1 or HDL-C levels in overweight/obese GDM patients. Because of the complex disturbance of metabolic parameters in overweight/obese patients with diabetes as compared to the nonobese subjects, the genotype-related effects on the levels of apo AI or HDL-C and TG might be masked in overweight/obese patients. This might be similar to our previous observations showing that variants of apoL-I gene are associated with TG, apoC-III, and apoE levels in nonobese Chinese, but not in overweight/obese subjects because in obesity, the genotype effects on lipid levels might be masked by obesity-associated metabolic abnormalities (39).

We have additionally analyzed our data using regression analysis with the biomarkers as the outcomes and allelic variants, maternal BMI and glycemic status as predictors/confounders. The results demonstrated that the glycemic status and/or maternal BMI are important predictors/confounders of fasting Ins, TG, TC, LDL-C and HDL-C levels, whereas the allelic variants are not (**Supplementary Table 3**). These data suggest that the GALNT2 genetic variation are not significantly associated with metabolic abnormality in GDM pregnancy and metabolic adaption in normal pregnancy. These results are in contrast with the report of the GALNT2 variants are linked to variations of metabolic traits such as serum HDL-C and TG variations in non-pregnant subjects, such as in Mexican dyslipidemic study samples of Mexican, Mexican American and European populations using a GWAS approach (17).

Our study has some strengths and limitations. The strengths are as follows. First, the study spans maternal and fetal/neonatal data. Second, the study adds to a limited body of evidence regarding genetics of hyperglycemia detected in pregnancy. Third, we provide some insight into genetic regulator of lipid metabolism and its impact on the biomarkers of insulin resistance in human pregnancy. The study limitations include a lack of data on GALNT2 expression levels in the population with different genotypes for the GALNT2 SNPs, which would have been helpful for providing further insights into the mechanisms responsible for the genetic association. We limit our findings to the descriptive characteristics.

In conclusion, our study does not provide evidence in favor of *GALNT2* rs4846914 and rs2144300 SNPs being associated with GDM in Southwest Chinese women, but demonstrates that rs4846914 and rs2144300 polymorphisms in the GALNT2 gene are associated with blood pressure variations in nonobese GDM and AI in overweight/obese GDM patients. Moreover, the former site is also associated with fetal growth in overweight/obese GDM women, while the latter site affects apo AI levels and pregnancy weight gain in overweight/obese control women. Our results suggest that the rs4846914 and rs2144300 polymorphisms might be associated with maternal cardiovascular risk and neonatal growth in GDM women and thus a potentially increased future risk of type 2 diabetes and CHD in the mothers and their offspring. Screening for these variants in GDM patients with different BMI levels, as well as reducing overweight/obesity through changes in diet and lifestyle in women with GDM, might be beneficial and have preventive effects against complications in these patients.

## Data Availability Statement

The datasets for this study can be found here: GALNT2 (rs2144300 and rs4846914): (https://www.ncbi.nlm.nih.gov/snp/rs2144300; https://www.ncbi.nlm.nih.gov/snp/rs4846914).

## Ethics Statement

The study was carried out in accordance with the Declaration of Helsinki. Written informed consent was obtained from all participants. This study was approved by the Institutional Review Board of the West China Second University Hospital, Sichuan University (No. 2017–033).

## Author Contributions

HB conceived and designed the experiments, analyzed the data, and revised the paper. LG performed experiments and wrote the paper. XL was responsible for patient screening. MZ, YW, RL, and YL helped with the experiments. PF helped with the experiments and revised the paper. All authors contributed to the article and approved the submitted version.

## Funding

This study was supported by grants from the National Natural Science Foundation of China (39870749), the National Key Research and Development Program of China (2016YFC1000400), Program for Changjiang Scholars and Innovative Research Team in University (IRT0935), the Fundamental Research Funds for the Central Universities, and Research Seed Fund from West China Second University Hospital of Sichuan University (HB).

## Conflict of Interest

The authors declare that the research was conducted in the absence of any commercial or financial relationships that could be construed as a potential conflict of interest.
