# Supplementary material for: Maternal GALNT2 Variations Affect Blood Pressure, Atherogenic Index, and Fetal Growth, Depending on BMI in Gestational Diabetes Mellitus
Source: Front Endocrinol (Lausanne). 2021 Jun 29;12:690229. doi: 10.3389/fendo.2021.690229 (PMC8276310; doi:10.3389/fendo.2021.690229)
Supplement: Supplementary file 1 [file Table_1.docx]

**S Table 1. Clinical characteristics and metabolic profile of GALNT2 rs4846914 genotypes in GDM patients and controls**

|  | GDM | | |  | Control | | |
| --- | --- | --- | --- | --- | --- | --- | --- |
|  | GG(271) | GA(156) | AA(34) |  | GG(366) | GA(217) | AA(43) |
| **Clinical characteristics** |  |  |  | |  |  |  |
| Age(years) | 35.92±3.39 | 34.05±4.37 | 34.78±4.30 | | 35.13±4.08 | 34.87±4.48 | 34.73±4.24 |
| Gestation age(weeks) | 39.04±0.96 | 39.03±0.84 | 39.15±0.68 | | 39.30±0.89 | 39.13±1.11 | 39.21±0.89 |
| Prepregnancy BMI (kg/m^2^) | 22.36±3.13 | 22.22±3.83 | 22.20±2.89 | | 21.2±3.45 | 21.22±2.42 | 20.77±2.84 |
| Weight gain during pregnancy (kg) | 10.61±4.22 | 12.15±4.09 | 11.84±4.39 | | 13.8±4.47 | 13.61±3.92 | 14.43±5.00 |
| Delivery BMI (kg/m^2^) | 27.36±5.98 | 26.94±3.96 | 26.83±2.87 | | 26.67±3.91 | 26.51±2.61 | 26.46±2.93 |
| Neonatal birth height (cm) | 49.53±3.45 | 49.77±1.75 | 49.00±1.84 | | 49.94±2.00 | 49.68±1.72 | 49.67±1.52 |
| Neonatal birth Weight (g) | 3363.91±495.37 | 3372.20±437.10 | 3297.29±352.44 | | 3468.91±469.92 | 3357.70±402.03 | 3361.94±419.13 |
| SBP(mmHg) | 116.02±9.54 | 116.04±10.89 | 115.1±12.07 | | 113.61±9.71 | 113.71±10.22 | 114.94±10.27 |
| DBP(mmHg) | 75.30±9.43 | 73.45±9.77 | 72.52±8.90 | | 71.26±7.59 | 71.84±9.08 | 72.57±7.90 |
| **Metabolic profile** |  |  |  | |  |  |  |
| Fasting Ins (pmol/L) | 82.97±56.29 | 86.74±57.22 | 95.30±64.59 | | 71.47±41.16 | 68.93±36.30 | 78.23±40.15 |
| Fasting Glu (mmol/L) | 4.43±0.65 | 4.44±0.78 | 4.40±0.78 | | 4.24±0.67 | 4.15±0.60 | 4.18±0.61 |
| HOMA-IR | 3.13±2.64 | 3.30±3.58 | 2.92±3.96 | | 2.20±1.32 | 1.92±1.22 | 2.12±1.89 |
| Triglycerides（mmol/L） | 3.50±1.06 | 3.95±1.69 | 3.94±1.83 | | 3.54±1.30 | 3.57±1.42 | 3.66±1.49 |
| TC（mmol/L） | 5.78±0.93 | 5.95±1.02 | 5.97±1.18 | | 5.89±1.28 | 6.04±1.19 | 6.04±1.15 |
| HDL-C（mmol/L） | 1.99±0.53 | 1.92±0.43 | 2.00±0.49 | | 2.02±0.53 | 1.98±0.44 | 2.01±0.44 |
| LDL-C（mmol/L） | 2.93±0.77 | 3.02±1.17 | 2.97±0.92 | | 2.99±1.09 | 3.19±0.99 | 3.15±1.06 |
| non-HDLC （mmol/L） | 3.98±0.78 | 4.09±0.93 | 4.13±1.38 | | 3.95±1.05 | 4.07±1.08 | 4.10±1.13 |
| Atherogenic index | 2.06±0.80 | 2.24±0.85 | 2.09±0.66 | | 1.92±0.61 | 2.09±0.66 | 2.06±0.69 |
| TG/HDL-C | 1.99±0.90 | 2.45±2.12 | 2.14±1.10 | | 1.79±0.85 | 1.87±0.8 | 1.95±0.94 |
| apoA1(g/L) | 2.26±0.30 | 2.30±0.38 | 2.30±0.38 | | 2.43±0.40 | 2.38±0.42 | 2.45±0.48 |
| apoB(g/L) | 1.12±0.22 | 1.18±0.25 | 1.18±0.28 | | 1.13±0.31 | 1.17±0.27 | 1.16±0.28 |
| apoB/apoA1 ratio | 0.54±0.12 | 0.56±0.13 | 0.57±0.14 | | 0.53±0.18 | 0.56±0.15 | 0.54±0.16 |
