# Supplementary material for: Maternal GALNT2 Variations Affect Blood Pressure, Atherogenic Index, and Fetal Growth, Depending on BMI in Gestational Diabetes Mellitus
Source: Front Endocrinol (Lausanne). 2021 Jun 29;12:690229. doi: 10.3389/fendo.2021.690229 (PMC8276310; doi:10.3389/fendo.2021.690229)
Supplement: Supplementary file 2 [file Table_2.docx]

1. **Table 2. Clinical characteristics, hormonal levels and metabolic profile of GALNT2 rs2144300 genotypes in GDM patients and controls**

|  | GDM | | |  | Control | | |
| --- | --- | --- | --- | --- | --- | --- | --- |
|  | CC(308) | CT(142) | TT(11) |  | CC(415) | CT(183) | TT(28) |
| **Clinical characteristics** |  |  |  | |  |  |  |
| Age(years) | 34.79±4.26 | 34.1±4.35 | 37.07±3.20 | | 34.70±4.26 | 34.94±4.31 | 35.14±4.69 |
| Gestation age(weeks) | 39.03±0.94 | 39.04±0.85 | 39.32±0.56 | | 39.29±0.87 | 39.10±1.12 | 39.38±1.11 |
| Prepregnancy BMI (kg/m^2^) | 22.30±2.93 | 22.1±3.85 | 21.68±2.32 | | 20.82±2.79 | 21.34±2.79 | 20.54±2.55 |
| Weight gain during pregnancy (kg) | 11.77±4.46 | 11.89±4.05 | 11.79±3.23 | | 14.31±4.85 | 13.61±4.10 | 14.04±4.67 |
| Delivery BMI (kg/m^2^) | 26.90±2.91 | 26.94±4.75 | 26.32±2.78 | | 26.46±2.88 | 26.62±3.14 | 26.07±2.55 |
| Neonatal birth height (cm) | 49.50±2.02 | 49.58±3.33 | 49.53±1.82 | | 49.92±1.97 | 49.64±1.73 | 49.80±1.47 |
| Neonatal birth Weight (g) | 3373.76±485.10 | 3327.50±435.96 | 3430.00±360.40 | | 3456.54±502.42 | 3355.03±396.8 | 3411.33±409.32 |
| SBP(mmHg) | 115.08±11.95 | 115.57±10.51 | 116.07±11.15 | | 114.65±10.09 | 113.88±10.11 | 112.00±10.28 |
| DBP(mmHg) | 72.15±9.05 | 73.54±9.45 | 75.71±8.97 | | 72.21±8.82 | 71.66±7.89 | 69.93±6.69 |
| **Metabolic profile** |  |  |  | |  |  |  |
| Fasting Ins (pmol/L) | 84.51±57.91 | 85.29±55.97 | 96.76±58.75 | | 72.19±47.37 | 68.88±35.03 | 73.77±42.28 |
| Fasting Glu (mmol/L) | 4.43±0.98 | 4.38±0.80 | 4.45±0.77 | | 4.20±1.08 | 4.07±0.82 | 4.24±0.80 |
| HOMA-IR | 3.81±11.85 | 3.47±6.75 | 3.20±2.05 | | 2.41±3.29 | 2.35±3.87 | 2.06±1.31 |
| Triglycerides（mmol/L） | 3.93±1.78 | 3.86±1.44 | 3.13±1.05 | | 3.65±1.47 | 3.60±1.43 | 3.35±1.39 |
| TC（mmol/L） | 6.01±1.46 | 5.95±0.96 | 5.56±1.06 | | 6.05±1.14 | 6.02±1.27 | 5.83±1.25 |
| HDL-C（mmol/L） | 2.00±0.48 | 1.96±0.43 | 1.79±0.47 | | 2.03±0.44 | 1.94±0.43 | 1.95±0.57 |
| LDL-C（mmol/L） | 2.97±0.92 | 3.02±1.16 | 2.85±0.74 | | 3.14±1.05 | 3.20±1.05 | 2.93±0.98 |
| non-HDLC （mmol/L） | 4.10±1.34 | 4.04±0.90 | 4.21±0.63 | | 4.04±1.12 | 4.20±1.10 | 3.78±0.85 |
| Atherogenic index | 2.09±0.64 | 2.14±0.71 | 2.36±0.78 | | 2.01±0.69 | 2.16±0.63 | 1.99±0.73 |
| TG/HDL-C | 2.09±0.64 | 2.18±1.28 | 2.08±1.02 | | 2.01±0.69 | 1.87±0.86 | 1.83±1.03 |
| apoA1(g/L) | 2.29±0.38 | 2.31±0.36 | 2.19±0.36 | | 2.45±0.48 | 2.35±0.41 | 2.44±0.44 |
| apoB(g/L) | 1.18±0.28 | 1.18±0.23 | 1.12±0.29 | | 1.16±0.28 | 1.18±0.29 | 1.12±0.29 |
| apoB/apoA1 ratio | 0.57±0.14 | 0.55±0.12 | 0.60±0.170 | | 0.53±0.16 | 0.58±0.15 | 0.52±0.21 |
