# Supplementary material for: Maternal GALNT2 Variations Affect Blood Pressure, Atherogenic Index, and Fetal Growth, Depending on BMI in Gestational Diabetes Mellitus
Source: Front Endocrinol (Lausanne). 2021 Jun 29;12:690229. doi: 10.3389/fendo.2021.690229 (PMC8276310; doi:10.3389/fendo.2021.690229)
Supplement: Supplementary file 3 [file Table_3.docx]

S. Table 3. Association of important independent predictors/confounders with the biomarkers as the outcomes by multivariate regression analysis in the women with and without GDM

| Independents | Unstandardized coefficients | | Standardized coefficients | *t* | *p* |
| --- | --- | --- | --- | --- | --- |
|  | *β* | S.E.M. | *β* |  |  |
| Model Ⅰ: Fasting insulin level as dependent variable | | | | | |
| Constant | 11.147 | 0.624 |  | 17.857 | 0.000 |
| Glycemic status | 3.539 | 0.953 | 0.112 | 3.714 | 0.000 |
| Maternal BMI (kg/m^2^) | 0.461 | 0.151 | 0.093 | 3.064 | 0.002 |
| Model Ⅱ: Triglyceride level as dependent variable | | | | | |
| Constant | 2.404 | 0.402 |  | 5.974 | 0.000 |
| Maternal BMI (kg/m^2^) | 0.046 | 0.015 | 0.092 | 3.048 | 0.002 |
| Glycemic status | 0.227 | 0.094 | 0.073 | 2.404 | 0.016 |
| Model Ⅲ: LDL-C level as dependent variable | | | | | |
| Constant | 4.131 | 0.268 |  | 15.442 | 0.000 |
| Maternal BMI (kg/m^2^) | -0.037 | 0.010 | -0.112 | -3.708 | 0.000 |
| Glycemic status | -0.158 | 0.063 | -0.067 | -2.517 | 0.012 |
| Model Ⅳ: TC level as dependent variable | | | | | |
| Constant | 7.181 | 0.324 |  | 22.155 | 0.000 |
| Maternal BMI (kg/m^2^) | -0.047 | 0.012 | -0.110 | -3.653 | 0.000 |
| Model Ⅴ: HDL-C level as dependent variable | | | | | |
| Constant | 2.545 | 0.119 |  | 21.422 | 0.000 |
| Maternal BMI (kg/m^2^) | -0.021 | 0.004 | -0.141 | -4.658 | 0.000 |

Glycemic status ( GDM＋, GDM－)
